# Perinatal ampicillin exposure alters murine maternal fecal bile acid and acylcarnitine profiles

**DOI:** 10.1080/19490976.2026.2690698

**Published:** 2026-06-29

**Authors:** Simone Zuffa, Sydney P. Thomas, Abubaker Patan, Ipsita Mohanty, Yasin El Abiead, Victoria Deleray, Kine Eide Kvitne, Armin Kousha, Emi Suzuki, Chih Ming Tsai, Griffith Nguyen, Benjamin Ho, George Y. Liu, Victor Nizet, Pieter C. Dorrestein, Fatemeh Askarian, Shirley M. Tsunoda

**Affiliations:** a Skaggs School of Pharmacy and Pharmaceutical Sciences, University of California San Diego, La Jolla, CA, USA; b Collaborative Mass Spectrometry Innovation Center, University of California San Diego, La Jolla, CA, USA; c Department of Pediatrics, University of California San Diego, La Jolla, CA, USA; d Department of Gastroenterology, Rady Children's Hospital San Diego, San Diego, CA, USA; e Division of Infectious Diseases, Department of Pediatrics, University of California San Diego, La Jolla, CA, USA; f Glycobiology Research and Training Center, University of California San Diego, La Jolla, CA, USA

**Keywords:** Ampicillin, pregnancy, untargeted metabolomics, microbiome, antibiotics

## Abstract

Maternal intrapartum antibiotic prophylaxis (IAP) and postpartum maternal antibiotic usage are increasingly common and have been linked to altered growth and immune development in offspring. However, the mechanisms underlying these effects, particularly those arising from indirect early-life exposure to antibiotics, remain poorly understood. Here, using a preclinical murine model, we examined the impact of *in vivo* antepartum and postpartum maternal ampicillin administration on the maternal fecal microbiome and metabolome. Ampicillin treatment resulted in a significant depletion of bacterial species belonging to the *Muribaculaceae* family, including *Muribaculum intestinale* and *Duncaniella dubosii*, accompanied by a cohort-dependent enrichment of *Enterococcus* and *Prevotella* species. These microbial shifts coincided with substantial and reproducible metabolic remodeling, including elevated fecal acylcarnitines and altered bile acid profiles. Notably, we identified two previously uncharacterized trihydroxylated bile acids conjugated to a hexose moiety, which we annotated as cholic acid-galactose and taurocholic acid-galactose and synthesized. These metabolites were consistently associated with antibiotic exposure across public metabolomics data repositories. Finally, alterations in the maternal fecal microbiome and metabolome were associated with increased weight gain in offspring, suggesting potential pathways by which maternal antibiotic exposure may influence early developmental outcomes. These findings highlight microbial and metabolic signatures linked to perinatal antibiotic use and underscore the need to balance infection control with long-term infant health considerations.

## Introduction

Birth is a defining moment in infant development. During and after delivery, microorganisms from the mother and surrounding environment colonize the newborn^1^ ^,^ ^2^, and initiate molecular interactions that are fundamental for immune maturation^3^ ^,^ ^4^, neurodevelopment^5-7^, and numerous other biological processes.^8^ ^,^ ^9^ Disruptions to these early gut microbial communities, particularly via external factors such as antibiotic exposure^10-12^, have been associated with increased risks for several later-life conditions, such as obesity^13^ ^,^ ^14^, type 1 diabetes^15^, atopic diseases^16^ ^,^ ^17^, and neurodevelopmental disorders.^18^ ^,^ ^19^ Despite growing awareness of these risks, the impact of indirect infant exposure to antibiotics via maternal administration remains poorly understood. Importantly, prophylactic antibiotic treatments before or during labor are commonly used to reduce complications and infections in both mothers and neonates across high- to low- and middle-income countries (LMICs).^20-22^ Notably, 92% of maternal deaths happen in LMICs according to the WHO, with postpartum infections being one of the major causes.^23^ As such, the consequences of these interventions require deeper investigation.

Ampicillin, a broad-spectrum *β*-lactam antibiotic, is commonly administered intravenously to at-risk mothers as intrapartum antibiotic prophylaxis (IAP) to prevent early-onset group B *Streptococcus* (GBS) disease in newborns.^24^ Approximately 20% to 30% of pregnant women test positive for GBS during routine screening in late pregnancy^25^ and about 0.2 cases per 1,000 live births result in early-onset invasive infections in the US, according to the CDC.^26^ Although IAP significantly reduces the risk of early-onset disease (EOD), GBS remains a leading cause of neonatal sepsis and meningitis, with a mortality rate of ~5% among affected infants.^27^ EOD survivors are also at greater risk of neurodevelopmental disorders including cerebral palsy, blindness, deafness, and cognitive delay.^27^ Importantly, IAP does not reduce the incidence of late-onset GBS disease.^28^ Furthermore, IAP has been shown to disrupt microbial colonization in neonates, possibly impairing the timing of early-life microbiota assembly.^29^ ^,^ ^30^ Finally, a large retrospective study on more than 200,000 children linked GBS-IAP with sustained increases in infant body mass index (BMI) from 6 months to 5 y of age, compared to no antibiotic exposure.^31^ These observations underscore the need to better understand how perinatal antibiotic administration, particularly the timing of exposure, modulates the maternal gut microbiome and metabolome composition, and its downstream influence on offspring development.

To study this, we investigated in a preclinical murine model the *in vivo* effects of maternal exposure to ampicillin, administered either antepartum or postpartum, on maternal gut microbial and metabolic profiles via shotgun metagenomics sequencing and untargeted liquid chromatography coupled with tandem mass spectrometry (LC-MS/MS) metabolomics. Notably, while fecal microbiome alterations appeared to mostly be cohort-dependent, we observed significant and reproducible alterations in the acylcarnitine and bile acid profiles across cohorts. These metabolites, known to play a critical role in energy homeostasis and immune regulation^32^ ^,^ ^33^, have the potential to influence offspring physiology through maternal transfer.^34^ ^,^ ^35^ Both antepartum and postpartum ampicillin exposure were associated with increased offspring weight and the antepartum treatment resulted in more persistent alterations to the maternal fecal metabolome. These findings suggest that the timing of perinatal ampicillin exposure can differentially shape fecal maternal microbiome and metabolome in mice, with possible implications for neonatal development. Our results emphasize the need to critically evaluate the necessity, spectrum, and timing of prophylactic antibiotic administration surrounding childbirth.

## Materials and methods

### Animal study and sample collection

Wild-type female timed pregnant C57BL/6 mice (strain#000664) were either obtained from the Jackson laboratory at gestational day (GD) 12 or 13 or manually set up for mating for 24 hours using 10- to 12-week-old male and female mice (1:1). Following confirmation of pregnancy on GD14, determined by abdominal palpation and visible enlargement of the abdomen, the animals were randomized into the designated experimental treatments and assigned to one of two cohorts: the Antepartum cohort or the Postpartum cohort. In the Antepartum cohort, animals were administered 150 mg/kg of ampicillin (AMP) or phosphate-buffered saline (PBS) intravenously via retro-orbital injection on GD17 and GD18, with delivery predicted on GD19. In the Postpartum cohort, animals received the same retro-orbital injections of AMP or PBS on postnatal days 2 and 3 (PND2 and PND3). All mice were anesthetized with isoflurane prior to intravenous administration. The 150 mg/kg AMP dose was determined according to the following criteria. In humans, the standard IAP regimen with ampicillin is 2 g IV initially, followed by 1 g IV every 4 hours until delivery, according to the guidance of the American College of Obstetricians and Gynecologists. This corresponds to 8 g of AMP during the first 24 hours, and 6 g per d subsequently. For an average 27.5-y-old pregnant woman, weighing 60–80 kg, this equates to 100–133 mg/kg during 24 hours. IAP is recommended to start at least 4 hours before delivery, and first-time labors typically last 12–18 hours. Importantly, mice exhibit faster drug clearance than humans as the half-life of ampicillin in mice is ~20 minutes compared to ~1–2 hours in humans (IARC Working Group on the Evaluation of Carcinogenic Risks to Humans). Thus, an AMP dose of 150 mg/kg falls within a clinically relevant range as we also aimed to induce a measurable perturbation of the maternal microbiome and metabolome within a time-compressed life history, given the short gestation and peripartum period in mice. Animals were single-housed in filter-top cages and had *ad libitum* access to a chow diet (2020X Teklad Global Soy Protein-Free Extruded Rodent Diets, inotiv/ENVIGO) and water. Ambient temperature was maintained at 20–22 °C, humidity at 30–70%, and a 12 hour light/12 hour dark cycle was kept throughout the experiment. Fecal pellets were collected using sterile tweezers at multiple timepoints and immediately stored at −80 °C. The attempt to collect milk and blood from the pups resulted in a high level of cannibalism. To measure offspring weight at PND21, the study was then repeated with minimal handling and no sample collection. All experiments were conducted under approval of the Institutional Animal Care and Use Committee, UC San Diego (IRB protocols S00227M and S18200). Collected fecal pellets were thawed and equally split into two aliquots: one for untargeted metabolomics and one for shotgun metagenomic sequencing. Samples for untargeted metabolomics were extracted using 10 μL of cold 50% (v/v) methanol (MeOH) per 1 mg of fecal pellet. Following the addition of the extraction solvent, samples were homogenized using a 5 mm stainless steel bead in a TissueLyser II (QIAGEN) for 5 minutes at 25 Hz and then incubated at 4 °C for 30 minutes. Samples were subsequently centrifuged at 21,130 *g* for 3 minutes, and supernatants were collected in a 96-well plate for untargeted metabolomics analysis.

### Untargeted metabolomics – data acquisition

After randomization, samples were injected in an untargeted metabolomics analysis platform comprising an UltiMate 3000 LC system (Thermo Fisher Scientific) coupled to a Q-Exactive Orbitrap mass spectrometer (Thermo Fisher Scientific) as previously described.^36^ Briefly, chromatographic separation was performed using a Kinetex C18 column (Phenomenex) and two mobile phases: solvent A (water + 0.1% formic acid) and solvent B (acetonitrile + 0.1% formic acid). Flow rate was set at 0.5 mL/minutes and the following linear gradient was used: 0–1 minutes, 5% B; 1–7 minutes, 98% B; 7–7.5 minutes, 98% B; 7.5–8 minutes, 5% B; and 8–10 minutes, 5% B. Data-dependent acquisition (DDA) mode was used to acquire MS/MS data in positive electrospray ionization (ESI+). The mass spectrometry scan range was programmed to 100–1500 *m/z* with a resolution at *m/z* 200 set to 35,000 with 1 microscan. Automatic gain control (AGC) was set to 5E4 with a maximum injection time of 100 ms. Up to 5 MS2 (TopN = 5) spectra per MS1 were acquired with a resolution at *m/z* 200 set to 35,000 with 1 microscan and AGC target of 5E4. An isolation window of 3.0 *m/z*, a stepwise normalized collision energy (20, 30, and 40 eV), and a dynamic exclusion of 10 seconds were used.

### Untargeted metabolomics – data processing

Experimental data were converted into an open-access format (.mzML) using ProteoWizard MSConvert^37^ and uploaded to GNPS/MassIVE (MSV000089558 and MSV000092652). Batch processing via MZmine 4.1^38^ was used to detect and extract metabolic features. Batch files can be downloaded from the associated GitHub page. Briefly, mass detection was performed on ions acquired between 0 and 10 minutes, with MS1 and MS2 noise levels set to 5E4 and 1E3, respectively. Parameters for the chromatogram builder were: five minimum consecutive scans, 1E5 minimum absolute height, and 10 ppm *m/z* tolerance. Local minimum resolver was applied after smoothing with the following parameters: chromatographic threshold 85%, minimum search range retention time 0.2 minutes, minimum ratio of peak top/edge 1.7. Following this, 13 C isotope filter and an isotope finder were used. A join aligner with weight for *m/z* set to 80 and retention time tolerance set to 0.2 minutes was used for feature alignment. If a feature was not detected in at least 2 samples, it was removed before using the peak finder. MetaCorrelate and ion identity networking were used before extracting the feature table. Additionally, the .mgf files necessary for feature-based molecular networking (FBMN)^39^, performed in GNPS2^40^, and molecular class prediction via CANOPUS^41^, performed in SIRIUS 6.1^42^, were generated. FBMN parameters were the following: 0.02 for precursor and fragment ion tolerances; 0.7 minimum cosine score, and 5 minimum matching peaks. The propagated candidate bile acid library^43^ was used to generate putative Level 2 annotations via MS/MS spectral matching, which were validated for the presence of diagnostic MS/MS fragment ions using MassQL^44^ bile acid-specific queries for the different bile acid steroid core hydroxylated statuses (https://massqlpostmn.gnps2.org/).^45^ The following queries were used:

• *Monohydroxylated* - QUERY scaninfo(MS2DATA) WHERE MS2PROD = 341.28:TOLERANCEMZ = 0.01:INTENSITYPERCENT = 5 AND MS2PROD = 323.27:TOLERANCEMZ = 0.01:INTENSITYPERCENT = 5
• *Dihydroxylated* - QUERY scaninfo(MS2DATA) WHERE MS2PROD = 339.27:TOLERANCEMZ = 0.01:INTENSITYPERCENT = 5 AND MS2PROD = 321.26:TOLERANCEMZ = 0.01:INTENSITYPERCENT = 5
• *Trihydroxylated* - QUERY scaninfo(MS2DATA) WHERE MS2PROD = 337.25:TOLERANCEMZ = 0.01:INTENSITYPERCENT = 5 AND MS2PROD = 319.24:TOLERANCEMZ = 0.01:INTENSITYPERCENT = 5
• *Tetrahydroxylated* - QUERY scaninfo(MS2DATA) WHERE MS2PROD = 335.24:TOLERANCEMZ = 0.01:INTENSITYPERCENT = 5 AND MS2PROD = 317.23:TOLERANCEMZ = 0.01:INTENSITYPERCENT = 5
• *Pentahydroxylated* - QUERY scaninfo(MS2DATA) WHERE MS2PROD = 333.22:TOLERANCEMZ = 0.01:INTENSITYPERCENT = 5 AND MS2PROD = 315.21:TOLERANCEMZ = 0.01:INTENSITYPERCENT = 5

Metabolic features were traced across the two cohorts using classical molecular networking with Min Cluster Size set to 0. Matching pairs were obtained after filtering the merged_pairs.tsv for differences in parent mass < 0.02, differences in retention time (RT) < 0.3 minutes, modified cosine score > 0.7, and removing matches within the same cohort. Networking jobs are available for download at the following links:

• Antepartum cohort - https://gnps2.org/status?task=80df966e597544b5b798d8571e7a1352
• Postpartum cohort - https://gnps2.org/status?task=d00e383149bf49a0b037ccbc399ab6c5
• Cross-cohort network - https://gnps2.org/status?task=5b5f690d5c20443585f62c1183baa633

### Untargeted metabolomics – data analysis

Feature tables from untargeted metabolomics analyses were imported in R 4.5.2 (R Foundation for Statistical Computing, Vienna, Austria) for downstream data analyses. Data quality was checked by examining total extracted peak areas, sample internal standard (IS), and QCmix, a reference sample containing 6 standards and acquired every 10 samples throughout the run. Blank filtering was performed by removing features when the mean peak areas across all samples were not at least five times the one observed in blanks. Additionally, features with near-zero variance were removed before dimensionality reduction using the function nearZeroVar from ‘caret v. 7.0.-1’. Principal component analysis (PCA) and partial least squares discriminant analysis (PLS-DA) were performed after robust center log ratio transformation (rclr) via the package ‘vegan v 2.7-3’ and ‘mixOmics v 6.34.0’.^46^ ^,^ ^47^ The rclr transformation was chosen to address the inherent compositionality and high sparsity of untargeted metabolomics data. Furthermore, as fecal samples were processed without prior lyophilization or precise mass normalization, rclr effectively accounts for varying sample concentrations by focusing on the ratios between features rather than absolute intensities. PERMANOVA was used to evaluate group centroid separation after dimensionality reduction. Performances of the PLS-DA models were evaluated using leave-one-out (loo) cross-validation, where the number of permutations corresponds to the number of total samples. Models with a classification error rate (CER) < 0.5 are considered discriminatory. Variable importance (VIP) scores were extracted for each feature and features with VIPs > 1 were considered significant for discrimination. Upset plots were generated using the package ‘UpSetR v 1.4.0’.^48^ The natural log ratios of the significant features extracted from the PLS-DA models, with AMP-associated features at the numerator and PBS-associated features at the denominator, were plotted over time and significance was tested via repeated Welch's T-test followed by Benjamini‒Hochberg (BH) correction. Metabolomics data were integrated with metagenomics data via DIABLO (block_PLSDA).^49^ The model was built on samples for which both sequencing and metabolomics data were available retaining the top 20 most discriminant features for each omics block. Performance was evaluated using loo cross-validation and correlations >0.7 between the features were visualized via a circos plot.

### Bile acid annotation

To further investigate and annotate molecules of interest, a single fecal sample was reinjected multiple times in the Q-Exactive Orbitrap mass spectrometer system with different acquisition parameters. Data were both acquired in positive and negative ESI mode and with different resolutions (up to 140,000), AGC targets (up to 5E5), and in-source energies (0, 10, 50, 80 eV). Additionally, the same fecal sample was also analyzed on a Bruker timsTof Pro2 mass spectrometer coupled to an Agilent HPLC system. Chromatographic separation was performed on a reverse-phase HPLC C18 column (2.1 mm × 120 mm) using 0.1% formic acid in water (A) and 0.1% formic acid in acetonitrile (B) as the mobile phase. The chromatography ran on a 12 minutes gradient: 0-0.50 minutes 5% B, 1.10 minutes 25% B, 7.50 minutes 40% B, 8.50 minutes 99% B, 10.10 minutes 5% B, 12.00 minutes 5% B. The column compartment was kept at 40 °C and the sample injection volume was 3 μL with a flow rate of 0.5 mL per minute. Detection was performed in positive ESI mode using a DDA method with TIMS-MS-PASEF with the following parameters: nebulizer gas pressure at 2.2 bar, dry temperature 220 °C, gas flow 10 L/minutes. The mass range was collected from 20 *m/z* to 1300 *m/z*, the PASEF used a 100 ms ramp time and 100 ms accumulation time, and the 1/K_0_ ranges from 0.80 to 1.2 Vs/cm^2^. The raw data file was then imported into Metaboscape 2025b, where it was processed as a project with positive polarity and the T-ReX 4D (LC-TIMS-QTOF) workflow. The filter parameters included a minimum of 1 feature for extraction and presence in a minimum of 1 analysis. The default workflow methods and calibration methods available were used to process the features from the sample. Theoretical reference collision cross-section (CCS) values were predicted in the CCS-Predict Pro 2025 model.

### Bile acid synthesis

To synthesize cholic acid-galactose, 50 mg (1 equiv.) of cholic acid was dissolved in 3 mL of dry DMF (Dimethylformamide) in a 20 mL scintillation vial equipped with a magnetic stir bar. To this solution, 44 mg (1.2 equiv.) of EDC (3-{[(Ethylimino)methylidene]amino}-*N*,*N*-dimethylpropan-1-amine) was added, and the mixture was stirred at room temperature for 15–20 minutes to allow for activation. Subsequently, 44 mg (2 equiv.) of *α*-D-galactose and 8 mg (0.5 equiv.) of DMAP (*N*,*N*-Dimethylpyridin-4-amine) were added while stirring. The reaction mixture was stirred overnight at room temperature, and the progress was monitored by thin-layer chromatography. Upon completion, the solvent was removed by rotary evaporation, and the crude product was used for further analysis. To synthesize taurocholic acid-galactose, 13 mg (1 equiv.) of taurocholic acid was dissolved in 3 mL of toluene, and 100 mg (10 equiv.) of acetobromo-*α*-D-galactose methyl ester was added, followed by 13.9 mg (2 equiv.) of silver carbonate. The reaction mixture was refluxed and stirred at 75 °C for more than 24 hours. After reflux, the mixture was cooled to room temperature, filtered, and evaporated to dryness under vacuum. The resulting solid residue was dissolved in 10 mL of methanol, and 6 equivalents of 1 M aqueous LiOH were added. The mixture was stirred at room temperature for 28 hours. After the solvent removal, the product was neutralized with 0.5 mL of 1 M acetic acid. The reaction crude was used for further analysis.

### Shotgun metagenomics – sequencing

Aliquots of the fecal samples were transferred to the UC San Diego Microbiome Core to perform DNA extraction as previously described.^50^ Briefly, samples were purified via the MagMAX Microbiome Ultra Nucleic Acid Isolation Kit (Thermo Fisher Scientific) using a KingFisher Flex robot (Thermo Fisher Scientific). Blanks and mock communities (Zymo Research Corporation) were included in the analysis for quality control. DNA was quantified via a PicoGreen fluorescence assay (Thermo Fisher Scientific), and metagenomic libraries were prepared using the KAPA HyperPlus kit (Roche Diagnostics) following the manufacturer's instructions via an EpMotion automated liquid handler (Eppendorf). Shallow shotgun sequencing was performed on an Illumina NovaSeq 6000 platform with paired-end 150 bp cycles at the Institute for Genomic Medicine (IGM), UC San Diego.

### Shotgun metagenomics – data processing and analysis

Demultiplexed FASTQ files were imported in Qiita (Study ID # 15345) and processed using the default workflow for metagenomics data.^51^ Briefly, adapters and host genome (mouse genome GRCm39,

GCF_000001635.27) sequences were removed using qp-fastp-minimap2 2023.12.^52^ Then, qp-woltka 2024.09 was used to generate the operational genomic units (OGUs) table^53^, which taxonomy was derived via the Web of Life (WoL2) reference database.^54^ Finally, OGUs were filtered against Greengenes2 (gg/2024.09).^55^ The ‘phyloseq v 1.54.2’ package was used to manipulate the microbiome data.^56^ The median sequencing depth of the sample was ~2,000,000 reads and samples with less than 500,000 reads were discarded from downstream analysis. For alpha diversity analysis, OGUs table was rarefied to the minimum number of reads observed in a sample before calculating the Shannon diversity index. Before beta diversity analysis and differential abundance analysis, rare microbial features detected in less than 10% of the samples were removed. Additionally, a relative abundance filter was applied and OGUs accounting for <0.0001% of total reads in any given sample were excluded. After the applied filters, 597 distinct OGUs were retained and the lowest number of reads per OGU was 204. Differential OGUs of interest identified in the downstream analysis, such as *Muribaculum intestinale*, *Paramuribaculum intestinale*, and *Duncaniella dubosii,* were present across cohorts with 2,529,961, 1,321,040, and 1,111,263 reads, respectively. Additionally, a sensitivity analysis using a stricter 0.001% threshold was conducted and did not show any impact on beta diversity. The non-rarefied OGUs tables were robust center log ratio (rclr) transformed using the package ‘vegan v 2.7-3’ before PCA via ‘mixOmics v 6.34.0’.^46^ ^,^ ^47^ Features with near-zero variance were also removed before dimensionality reduction using the function nearZeroVar from ‘caret v. 7.0.-1’. Centroid separation was evaluated using PERMANOVA. Differential abundance analysis was performed using ‘ALDEx2 v 1.42.0’.^57^ Species were considered significantly different between groups if adjusted *p*-values after BH correction were <0.05. The natural log ratios of features associated with AMP exposure, summed at the numerator, or PBS, summed at the denominator, were plotted against time, as described for the metabolomics analysis. Overlap across the two cohorts was investigated via Upset plot.

### Data and code availability

Code used for the analysis and to generate the figures presented in this manuscript is available on GitHub (https://github.com/simonezuffa/Manuscript_AMP_Perinatal). Untargeted metabolomics data are publicly available in GNPS/MassIVE under the following accession codes: MSV000089558 (Antepartum cohort) and MSV000092652 (Postpartum cohort). Metagenomics data is available in Qiita (Study ID # 15345) and in ENA under the accession number PRJEB90218.

## Results

### Perinatal ampicillin exposure is associated with increased weight in offspring

**Figure 1.**
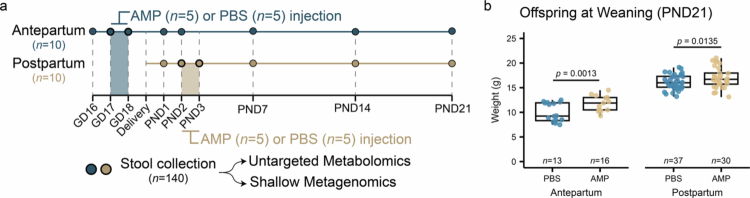
Perinatal maternal exposure to ampicillin is associated with increased offspring weight. (a) Two cohorts of dams (*n* = 20) were injected intravenously with either AMP (150  mg/kg) or PBS for 2 d either antepartum (GD17 and GD18) or postpartum (PND2 and PND3). Fecal samples (*n* = 131) were collected and analyzed via untargeted metabolomics and metagenomic sequencing. After quality control, 116 samples were included in the downstream analyses. (b) Offspring body weight was measured at weaning (PND21) in a subsequent, independent cohort. Offspring of dams exposed to AMP had a significantly higher weight compared to the respective controls in both treatments (*p* < 0.05). *P* values were obtained via estimated marginal means on a linear regression model adjusted for sex. Boxplots show the first (lower) quartile, median, and third (upper) quartile. Abbreviations: AMP, ampicillin; GD, gestational day; PND, postnatal day; PBS, phosphate-buffered saline.

Dams (*n* = 20) received retro-orbital intravenous injections of either 150 mg/kg of ampicillin (AMP) or phosphate-buffered saline (PBS) for two consecutive days. Treatments were administered either before the estimated delivery time (gestational day [GD]17 and GD18; Antepartum cohort) or after birth (postnatal day [PND]2 and PND3; Postpartum cohort). Fecal samples were collected 1 d prior to treatment, during treatment, and on PND7, PND14, and PND21, for untargeted metabolomics analysis via UPLC-MS/MS and shotgun metagenomic sequencing (Figure 1a). Sample collection was designed to capture key perinatal stages rather than to obtain identical timepoints across cohorts. In the Antepartum cohort, additional samples were collected at PND1 and PND2, given that the gut microbiome can undergo changes during delivery. The average litter size was 6.95 ± 1.32 (mean ± standard deviation). However, attempts to collect milk from dams and blood from pups resulted in a high incidence of cannibalism. To measure offspring weight at weaning (PND21), the study was therefore repeated with minimal handling besides treatment injections. The Antepartum and Postpartum cohorts then included four and ten dams, respectively. The average litter size was 7.35 ± 1.21 and no cannibalism was observed (Supplementary Table 1). Offspring body weight was recorded at weaning (PND21) and it was significantly higher (linear model with sex as covariate, *p* < 0.05 via estimated marginal means) in mice indirectly exposed to AMP during the perinatal period across both cohorts (Figure 1b).

### Perinatal ampicillin exposure disrupts the maternal fecal microbiome

**Figure 2.**
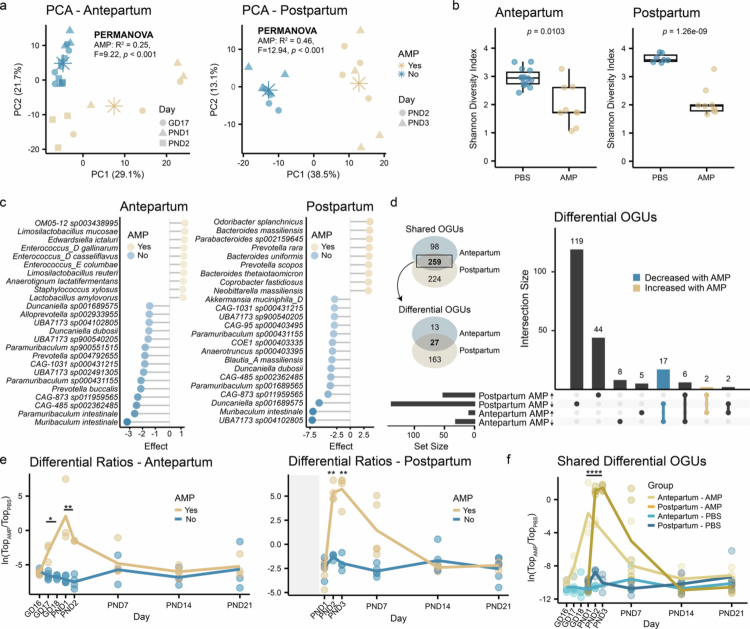
Perinatal ampicillin exposure alters the maternal fecal microbiome. (a) PCA of rclr transformed microbial features of fecal samples collected during treatment showed significant differences in response to AMP in both cohorts (PERMANOVA, *p* < 0.001). (b) Shannon alpha diversity was significantly reduced in dams receiving AMP (linear mixed effect model, all *p* < 0.05) and recovered at later timepoints (Supplementary Figure 1). (c) Top 10 enriched and top 15 depleted bacterial species in response to AMP, based on effect sizes obtained via ALDEx2. Only taxa with adjusted *p* < 0.05 are included. (d) Overlapping OGUs (*n* = 27) were altered in both cohorts following AMP treatment. Of these, 19 showed concordant alterations, either depleted (*n* = 17) or enriched (*n* = 2) in response to AMP in both cohorts, while 8 showed opposite trends (*e.g.* increased with AMP in one cohort while enriched with AMP in the other, and vice versa). Commonly depleted taxa included species belonging to *Muribaculum*, *Paramuribaculum*, and *Duncaniella* genera. AMP↑ indicated that the features were enriched with AMP, while AMP↓ indicated that the features were depleted with AMP and thus more abundant in PBS. Shared features include OGUs that did not pass FDR correction in the single models but they did when considering only the shared space. (e) Longitudinal log ratio analysis of differentially abundant species identified via ALDEx2 per cohort. Features enriched in response to AMP were summed at the numerator (Antepartum, *n* = 12; Postpartum, *n* = 85), whereas depleted features were summed at the denominator (Antepartum, *n* = 30; Postpartum, *n* = 181). The same number of features were considered at each timepoint. Significance was assessed using repeated Welch’s T-test with BH correction. (f) Log ratios of exclusively overlapping and directionally concordant species between cohorts across time (*n* = 19, 2 features at the numerator and 17 features at the denominator). Ratios in the Postpartum cohort were normalized to the mean ratio difference between the two cohorts before treatment. Top_AMP_ and Top_PBS_ indicate features enriched or depleted in response to AMP, respectively. Boxplots indicate the first (lower) quartile, median, and third (upper) quartile. Asterisks in PCA denote group centroids. Significance: *p < 0.05, **p < 0.01, ****p < 0.0001. Abbreviations: AMP, ampicillin; GD, gestational day; PND, postnatal day.

Metagenomic sequencing identified a time-dependent change in the maternal fecal microbiome throughout delivery and lactation (Supplementary Figure 1a) and alterations in response to AMP administration (PERMANOVA, *p* < 0.001) in both the Antepartum and Postpartum cohorts (Figure 2a). AMP significantly lowered Shannon alpha diversity (linear mixed effect model, all *p* < 0.05) during treatment (Figure 2b), which then recovered at later timepoints (Supplementary Figure 1b). A higher variability within the AMP groups was observed in both PCA and Shannon diversity analysis. Importantly, the Antepartum cohort presented 357 distinct OGUs, while the Postpartum cohort comprised 483 distinct OGUs after quality filtering (see Methods).

### Ampicillin consistently depletes bacterial species in the *Muribaculaceae* family

Differential abundance analysis using ALDEx2 identified 42 OGUs significantly altered by AMP in the Antepartum cohort after FDR correction (Supplementary Table 2). Specifically, species belonging to the *Muribaculaceae* family, including *Muribaculum intestinale, Paramuribaculum intestinale*, and *Duncaniella dubosii*, and to the *Prevotella*, *Alloprevotella, Parasutterella,* and *Alistipes* genera were depleted, whereas species belonging to the *Enterococcus*, *Limosilactobacillus*, and *Staphylococcus* genera, such as *Enterococcus_D gallinarum*, *Limosilactobacillus mucosae*, and *Staphylococcus xylosus*, were enriched in response to AMP (Figure 2c). In contrast, 266 OGUs were altered in the Postpartum cohort in response to the antibiotic after FDR correction (Supplementary Table 3). AMP induced enrichment of bacterial species belonging to the *Prevotella*, *Bacteroides*, *Escherichia*, *Phocaeicola*, *Alloprevotella*, and *Klebsiella* genera and the depletion of species belonging to the *Muribaculum*, *Duncaniella, Paramuribaculum*, *Blautia*, *Akkermansia,* and *Roseburia* genera (Figure 2c).

Commonly altered OGUs across the two cohorts were identified by first filtering the two distinct ALDEx2 outputs, before FDR correction, to retain only shared OGUs that were detected in both cohorts (*n* = 259). Then, the FDR correction was applied only on these subsets. As FDR was set at 0.05, the expected false positive rate (q^2^) was 0.0025 and the expected random overlap was 0.65 (0.0025 × 259). The observed significant overlap was of 27 OGUs (Supplementary Table 4). Interestingly, 19 OGUs showed concordant enrichment (*n* = 17) or depletion (*n* = 2) in response to ABX across the two cohorts, as alterations appeared to be largely cohort-dependent (Figure 2d). Bacteria depleted in both cohorts encompassed species belonging to the *Muribaculaceae* family, including *Muribaculum intestinale, Muribaculum gordoncarteri, Duncaniella dubosii, Paramuribaculum intestinale,* and the genera *UBA7173*, *CAG-873*, *CAG-1031*, and *CAG-485*. Only two species were commonly enriched after AMP administration, which included *Edwardsiella ictaluri* and the *Bacteroidaceae* species *OM05-12 sp003438995*. Analysis of log ratios between AMP-enriched OGUs summed at the numerator and AMP-depleted OGUs summed at the denominator revealed that disruption was transient. In the Postpartum cohort, effects were limited to the treatment period, whereas in the Antepartum cohort, effects persisted until PND2 (Figure 2e). Plotting the log ratios of exclusively overlapping OGUs between the two cohorts (*n* = 19), with concordant depletion or enrichment in response to AMP, recapitulated the observations obtained from the full comparisons (Figure 2f).

### Perinatal ampicillin exposure alters the maternal fecal metabolome

**Figure 3.**
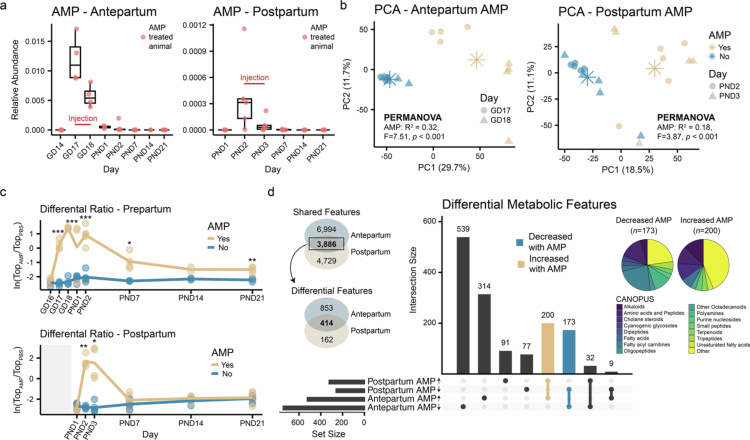
Perinatal ampicillin administration modifies maternal fecal metabolic profiles. (a) Relative peak area abundance of detected fecal ampicillin ([M + H] *m/z* 350.1174, RT 2.7 min) in treated dams. No AMP was administered at the earliest timepoints (GD14 - Antepartum and PND1 - Postpartum). (b) PCA of fecal samples collected during AMP exposure in the two cohorts. Fecal metabolic profiles were significantly altered based on exposure (PERMANOVA, *p* < 0.001). Two potential outliers were observed among AMP-treated animals in the Postpartum cohort. (c) Longitudinal analysis of the natural log ratios. Metabolic features increased with AMP were summed at the numerator (Antepartum, *n* = 1530; Postpartum, *n* = 618), whereas decreased features were summed at the denominator (Antepartum, *n* = 2003; Postpartum, *n* = 663). Features were obtained from PLS-DA models constructed using administration timepoints solely and filtered after using linear mixed effect models with BH correction. The same number of features were considered at each timepoint. Statistical significance was assessed using repeated Welch’s *T*-test with BH correction. (d) Overlapping metabolic features (*n* = 414) were altered in the two cohorts following AMP treatment. Of these, 373 showed concordant alterations, either increased (*n* = 200) or decreased (*n* = 173) in response to AMP. Molecular classes of concordant molecular features were predicted using CANOPUS. Classifications are reported in the pie charts. AMP↑ indicated that the features were enriched with AMP, while AMP↓ indicated that the features were depleted with AMP and thus more abundant in PBS. Boxplots show the first (lower) quartile, median, and third (upper) quartile. Asterisks in PCA represent group centroids. Significance: *p < 0.05, **p < 0.01, ***p < 0.001. Abbreviations: AMP, ampicillin; GD, gestational day; PND, postnatal day.

Untargeted metabolomics analysis of fecal samples confirmed AMP detection during administration and up to 2 d after the final injection (Figure 3a). Unsupervised PCA revealed time-dependent shifts in the fecal metabolome (Supplementary Figure 1c) and clear separation by treatment (PERMANOVA, *p* < 0.001) based on AMP exposure in both cohorts (Figure 3b), with a higher variability within the AMP group. The Antepartum cohort presented 10,880 metabolic features, while the Postpartum cohort comprised 8615 metabolic features after quality filtering (see Methods). Supervised PLS-DA models achieved near-perfect classification performance in both cohorts, highlighting the strong metabolic impact of AMP. Metabolic features driving group separation with VIP > 1 were extracted from both models and univariate analysis via linear mixed effect models was performed to calculate *p*-values and apply BH correction. A total of 3533 features were significantly altered (VIP > 1 and *p* adjusted < 0.05) in the Antepartum cohort in response to AMP (Supplementary Table 5), while 1281 were altered in the Postpartum cohort (Supplementary Table 6). The natural log ratios of the features that increased with AMP summed at the numerator and decreased with AMP summed at the denominator were analyzed over time (Figure 3c). Interestingly, the effect of AMP was detectable in the dams up to weaning (PND21) in the Antepartum cohort, whereas it was restricted to the administration period only in the Postpartum cohort. To trace molecular features of interest shared across the two cohorts, a cross-cohort molecular network without clustering between consensus MS/MS spectra was created. Network pairs were filtered to retain pairs with delta *m/z* < 0.02, delta retention time <0.3 min, and cosine similarity > 0.7. This returned an overlap of 3886 features and this shared metabolic space was then used to recalculate FDR, obtain the expected false positive rate (q^2^ = 0.0025), and calculate the expected random overlap (9.7, 0.0025 × 3886), as described for the microbiome analysis. The shared differential features across the two cohorts were 414 (Supplementary Table 7). Notably, 373 of them showed the same alterations in both cohorts, with 173 decreased and 200 increased in response to AMP treatment (Figure 3d). Feature annotation using FBMN, which is based on MS/MS spectral matching and represents a Level 2 annotation according to the Metabolomics Standard Initiative (MSI), and molecular class prediction via CANOPUS identified most as acylcarnitines, bile acids, polyamines, and small peptides.

### Fecal acylcarnitines and bile acids are consistently altered in response to ampicillin

**Figure 4.**
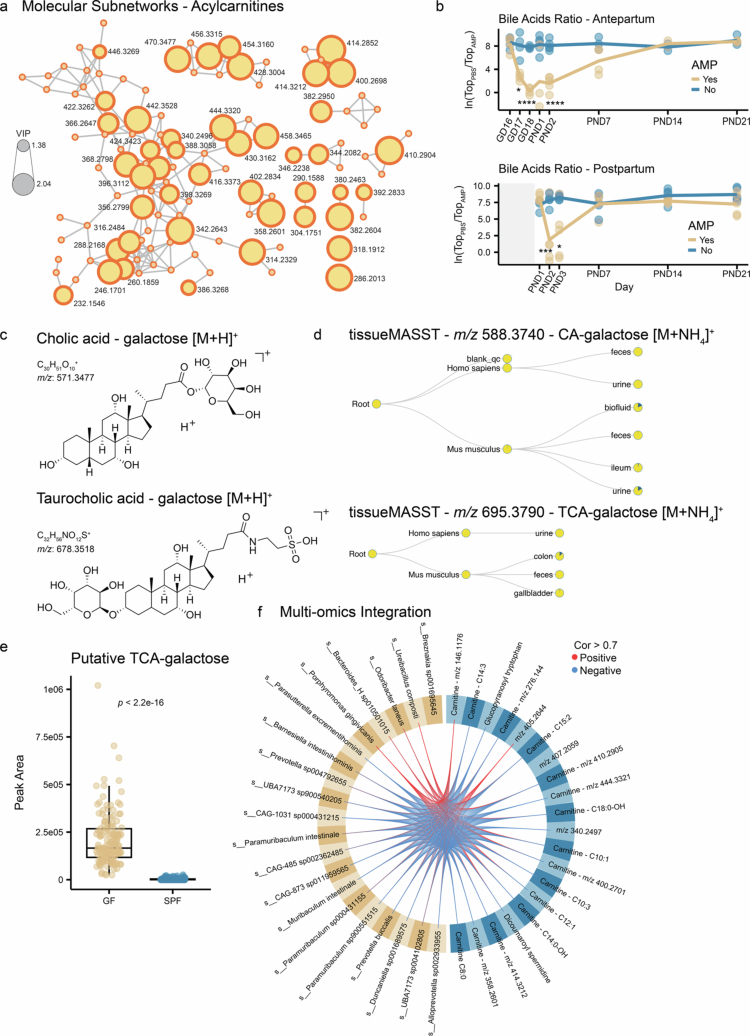
Fecal acylcarnitines and bile acids are altered by ampicillin treatment. (a) Submolecular network of acylcarnitines. Node size reflects VIP scores from the PLS-DA model. The majority of detected carnitines were enriched in response to AMP and putative annotations are provided in Supplementary Table 8. (b) Longitudinal analysis of the natural log ratios of bile acids altered by AMP across both cohorts. Bile acids increased with AMP (*n* = 2) were summed at the numerator, whereas the depleted ones (*n* = 8) were summed at the denominator. The same number of features were considered at each timepoint. AMP effect was restricted to the treatment period in the Postpartum cohort, while it persisted until PND2 in the Antepartum cohort. Statistical analysis was performed using repeated Welch’s T-test followed by BH correction. Significance: *p < 0.05, ***p < 0.001, ****p < 0.0001. (c) Structures of the synthetized cholic acid-galactose (C_30_H_51_O_10_, M + H) and taurocholic acid-galactose (C_32_H_56_NO_12_S, M + H). (d) tissueMASST output from MS/MS spectral searches of CA-galactose and TCA-galactose. The molecules were predominantly detected in murine samples. (e) Reanalysis of the external dataset (MSV000096038) in which tca-galactose was detected showed enrichment in GF animals compared to SPF ones (Mann–Whitney U test, *p* < 2.2e-16). (f) Circos plot obtained from multi-omics integration via DIABLO, combining metabolomics and metagenomics data in the Antepartum cohort. Red lines indicate positive correlations, and blue lines indicate negative correlations. Only correlations with a coefficient >0.7 are visualized. The model yielded a classification error rate (CER) of 0.16.

Several putatively annotated and predicted acylcarnitines were altered in maternal feces following AMP exposure (Figure 4a). In both cohorts, 43 distinct carnitines were consistently increased during antibiotic treatment. These included short and medium-chain carnitines, such as butyryl-carnitine (C4:0), valeryl-carnitine (C5:0), hexanoyl-carnitine (C6:0), octanoyl-carnitine (C8:0), decanoyl-carnitine (C10:0), as well as carnitines with longer chain length and/or hydroxyl groups, such as hydroxy-tetradecanoyl-carnitine (C14:0-OH). Through information propagation in the submolecular networks, CANOPUS class prediction, and MassQL validation of the diagnostic fragment ions for carnitines (*m/z* 60.0813 and *m/z* 85.0287), more than 30 carnitines were putatively annotated (Supplementary Table 8). No reference standards were used to confirm annotations through retention time matching, but retention time of the annotated acylcarnitines was consistent with the homologous series (Supplementary Figure 2). Importantly, bile acids profiles were also affected by AMP administration. In both cohorts, 10 different di-, tri-, and tetra-hydroxylated bile acids, which included the putatively annotated deoxycholic acid (DCA) and ketodeoxycholic acid (ketoDCA), were altered in response to AMP (Supplementary Table 9). The natural log ratios of these bile acids alone recapitulated the longitudinal observations obtained using all differential metabolic features (Figure 4b). We further investigated two unannotated bile acids (*m/z* 588.3740 and *m/z* 695.3790) consistently elevated in dams treated with AMP (Supplementary Figure 3a). These were trihydroxylated bile acids, putatively cholic acid (CA) and taurocholic acid (TCA), given the presence of the respective diagnostic ions of the steroid core and taurine (Supplementary Figure 3b). Interestingly, they were both ammonium adducts [M + NH4]+ , with the other ion forms being lower in abundance and failing to trigger MS/MS acquisition (Supplementary Figure 3c). SIRIUS chemical formula predictions returned C_30_H_50_O_10_ and C_32_H_55_NO_12_S, corresponding respectively to a cholic acid (C_24_H_40_O_5_) and taurocholic acid (C_26_H_45_NO_7_S ) conjugated to a hexose sugar (C_6_H_12_O_6_) via water loss. The spectra were further investigated using a timsTOF, where the [M + H]+ adduct of *m/z* 695.3790 returned a ΔCCS% of 5.8% relative to a theoretical TCA-hexose conjugate. Subsequently, we proceeded with the synthesis (see Methods for details) of both cholic acid-galactose and taurocholic acid-galactose (Figure 4c), whose reference spectra have now been added to the GNPS libraries, and obtained a Level 2 annotation via spectral matching (Supplementary Figure 3d).

The two spectra of the characterized bile acids were queried against the public metabolomics repositories via tissueMASST.^58^ These molecules appeared to be predominantly detected in mouse feces and gastrointestinal tract, and more rarely in human samples (Figure 4d). Interestingly, the unstructured search output obtained from tissueMASST showed detection across several murine datasets lacking metadata, but associated with germ-free conditions or antibiotics use (Supplementary Table 10). Reprocessing an external untargeted metabolomics dataset (*n* = 232) generated from the cecal content of germ-free (GF) or colonized (SPF) mice^58^, showed a significant enrichment of the putative TCA-galactose in GF animals (Figure 4e), confirming that these bile acid derivatives may serve as markers of altered microbial function or antibiotic exposure. Finally, multi-omics integration via DIABLO^49^, highlighted that both *Muribaculum intestinale* and *Paramuribaculum intestinale* had a strong negative correlation to several medium to long-chain carnitines, that we have previously shown to be enriched in response to AMP (Figure 4f).

## Discussion

The perinatal period, immediately before or after delivery, represents a critical window for the newborn's long-term development. As maternal microorganisms seed the infant at birth^59^, disruptions to maternal gut microbial communities or metabolic profiles may adversely affect the growth and immune developmental trajectories of offspring. In this study, we explored the impact of perinatal administration of ampicillin (AMP) on maternal fecal microbiome and metabolome in a mouse model. Notably, offspring indirectly exposed to AMP through maternal treatment exhibited increased weight at weaning (PND21) compared to unexposed controls. Direct exposure to broad-spectrum antibiotics, such as AMP, during the first months of life is already known to increase infant weight at 1 y of age or later in life.^13^ ^,^ ^31^ ^,^ ^60^ Nevertheless, further studies are required to elucidate these observations, as mechanisms remain elusive. It should also be noted that mice have larger offspring compared to humans and that their cecal microbiome contributes more substantially to nutrition and energy metabolism than in humans. Additionally, while mice were fed a plant-based diet (2020X Teklad Global) with minimal presence of soybean meal to reduce experimental variability caused by phytoestrogens, a degree of variability and differential impact of the gut microbiome could still be present due to the presence of complex polysaccharides. The use of defined or purified diets should be explored to further validate the results.

Across the two cohorts, AMP treatment resulted in clear disruptions to the maternal fecal microbiome composition. Interestingly, the proportion of overlapping altered bacterial species was low between the cohorts, suggesting that many observed changes were cohort-dependent. This variation could have been influenced by initial maternal colonization patterns or environmental factors. Nonetheless, some consistent findings emerged. We observed a reproducible depletion of species within the *Muribaculaceae* family, including *Muribaculum intestinale*, *Muribaculum gordoncarteri*, *Duncaniella dubosii*, *Paramuribaculum intestinale,* and the genera *UBA7173*, *CAG-873*, and *CAG-485*. Members of this family are involved in cross-feeding networks with other gut commensals such as *Bifidobacterium* and *Lactobacillus.* ^61^ Among them, *M. intestinale* has been shown to restrict pathogen colonization in mice by converting succinate to propionate^62^, a mechanism with potential relevance to early-life pathogen colonization resistance. Succinate itself has been shown to promote colonization by *Clostridium difficile* in mice^63^, suggesting that *M. intestinale* could play a protective role. Additionally, *M. intestinale* can produce a proinflammatory cardiolipin^64^, which may help prime immune development during early life. Similarly, *D. dubosii* has been associated with immunomodulatory effects via tryptophan metabolism in a murine model.^65^ In the Antepartum cohort, AMP also reduced the abundance of several *Alistipes* species, including *A. communis* and *A. timonensis,* which have reported immunomodulatory properties.^66^ In the Postpartum cohort, *Akkermansia muciniphila,* a mucin-degrading bacterium with protective effects against metabolic disorders and diet-induced obesity in mice^67^, was likewise depleted. Interestingly, early-life supplementation of *A. muciniphila* has been associated in humans with increased intestinal goblet cell numbers, reduced adiposity, and improved glucose homeostasis in adulthood.^68^ The depletion of these taxa in the dams during the perinatal period may reduce their transmission to offspring gastrointestinal tract, impairing the timing of early-life colonization and immune system development, and potentially increasing susceptibility to pathogen colonization.

The AMP treatment altered the maternal fecal metabolome in a more consistent way across the two cohorts. In both Antepartum and Postpartum cohorts, AMP administration resulted in elevated levels of multiple acylcarnitine species, including several previously uncharacterized molecules identified via molecular networking and MassQL. These molecules, important in host energy metabolism via *β*-oxidation^69^, have been previously reported to accumulate in the cecum, but not plasma, of antibiotic-treated mice.^70^ Acylcarnitines can be hydrolyzed by gut bacteria to carnitine and free fatty acids via acylcarnitine hydrolases (esterases).^71^ While fatty acids can then be used by bacteria for *β*-oxidation via the fatty acid degradation genes (*fad*)^72^, carnitine is metabolized by bacteria to trimethylamine (TMA) via the *cai* and *gbu* gene clusters^73^, which is then converted to TMAO by the host hepatic flavin-containing monooxygenase 3 (FMO3).^74^ Acylcarnitines have been shown to promote the growth of *Enterobacteriaceae* species in both human and rodents^71^, including opportunistic pathogens such as *Escherichia coli* and *Klebsiella* species, both of which were enriched following AMP treatment in the Postpartum cohort. These microorganisms are also known to develop resistance to AMP^75^ ^,^ ^76^ and they may be vertically transmitted to the infants during delivery or lactation. Similarly, in the Antepartum cohort, we observed enrichment of *Enterococcus* species, which are likewise capable of acquiring AMP resistance.^77^ ^,^ ^78^

The bile acid profiles were also affected by AMP. These steroidal molecules, derived from cholesterol metabolism, are closely regulated by gut microbial activity, as intestinal bacteria convert primary bile acids into secondary forms through diverse enzymatic pathways.^33^ ^,^ ^43^ We observed consistent depletion of microbially derived bile acids, including deoxycholic acid (DCA) and ketodeoxycholic acid (ketoDCA), consistent with previous reports.^79^ ^,^ ^80^ DCA, in particular, inhibits *Clostridium difficile* spore germination and vegetative growth in mice and *in vitro*.^81^ ^,^ ^82^ Thus, its depletion might facilitate pathogen colonization in offspring. Across both cohorts, we also observed a sustained enrichment of two previously uncharacterized bile acid conjugates: cholic acid- and taurocholic acid-hexose conjugates, likely containing galactose or potentially to one of its isomers. The detection of these two bile acid conjugates appeared to be relatively rare across public data but was mostly commonly observed along the murine gastrointestinal tract, as shown by tissueMASST.^58^ Their potential involvement in host-microbial co-metabolism was confirmed via observations in an external cohort of GF and SPF animals and no matches against any of the other domainMASSTs.^83-85^ Given the fecal enrichment of these bile acids in response to antibiotics and in GF condition, their origin might be host-derived and then metabolized by the gut microbiome. Nevertheless, the current biological role of these saccharide-conjugated bile acids remains unclear, and may reflect host-microbiome alterations in carbohydrate metabolism, a pathway that has also been linked to obesity and insulin resistance.^86^ As hexose represents a fundamental energy source for bacteria, these conjugates could be produced by the host to directly support the growth of microorganisms encoding for bile salt hydrolase/transferase genes (*bsh*). Additionally, excess monosaccharides have been associated with low-grade inflammation in murine models and conjugation/deconjugation of these molecules to bile acids by host-microbial metabolism could alter bioavailability. While fructose was shown to promote hepatic lipid accumulation^87^, galactose has been reported to activate immune cells.^88^ It is important to note that untargeted metabolomics analysis was conducted via reversed-phase (RP) liquid chromatography coupled with positive electrospray ionization (ESI+). While this approach is highly sensitive for the detection of non-polar and semi-polar species, such as the acylcarnitines and bile acid highlighted in this study, it inherently limits the detection of highly polar or negatively charged metabolites, such as small organic acids and sugars, which are better resolved using Hydrophilic Interaction Liquid Chromatography (HILIC) or negative mode ionization (ESI-).

## Conclusion

Our findings show that perinatal AMP exposure perturbs maternal microbiome and fecal metabolic profiles. Specifically, we observed reproducible changes in bile acid and energy metabolism. The contribution of these microbial and metabolic alterations are descriptive and their associations with the observed increase in offspring weaning weight remain hypothetical and will have to be further validated. However, these observations underscore the need for careful evaluation of the timing, necessity, and downstream consequences of broad-spectrum antibiotic use during the perinatal window.

## Limitations

The impact of indirect ampicillin exposure via maternal milk on offspring was not investigated due to challenges in sample collection from offspring and a high rate of cannibalism. Milking during the perinatal period exacerbated cannibalistic behavior, highlighting the need for careful evaluations of methodologies for sample collection during this crucial window. To measure the pup's weight at weaning, the study had to be repeated. The collection of low biomass samples from the offspring also posed a challenge, and appropriate negative controls should be rigorously implemented in future studies of this nature. Although the sample size was limited, the pronounced effect of the ampicillin treatment, coupled with longitudinal sampling and reproducibility across the two separate cohorts, strengthens the generalizability of our findings. It is important to acknowledge that observations from animal models may not always translate to humans. Untargeted metabolomics data were acquired in two separate batches, 1 y apart, which is suboptimal for direct comparisons. Nevertheless, our approach of first analyzing datasets independently and subsequently tracking molecular features of interest via high spectral similarity mitigates potential batch-related discrepancies. Metabolic feature annotations were obtained from MS/MS spectral matching against GNPS reference libraries, corresponding to level 2 annotation according to the Metabolomics Standard Initiative (MSI).

## Supplementary Material

Supplementary_Figures_V3.pdf Supplementary_Figures_V3.pdf

FigureS1.png FigureS1.png

supplemenatry_tables.xlsx supplemenatry_tables.xlsx

FigureS3.png FigureS3.png

FigureS2.png

## Data Availability

Untargeted metabolomics data are publicly available in GNPS/MassIVE under the following accession codes: MSV000089558 (Antepartum cohort) and MSV000092652 (Postpartum cohort). Metagenomics data is available in ENA under the accession number PRJEB90218.
