# Supplementary material for: Perinatal ampicillin exposure alters murine maternal fecal bile acid and acylcarnitine profiles
Source: Gut Microbes. 2026 Jun 29;18(1):2690698. doi: 10.1080/19490976.2026.2690698 (PMC13321893; doi:10.1080/19490976.2026.2690698)
Supplement: Supplementary_Figures_V3.pdf [file KGMI_A_2690698_SM2873.pdf]

Supplementary Figures

FIGURE S1

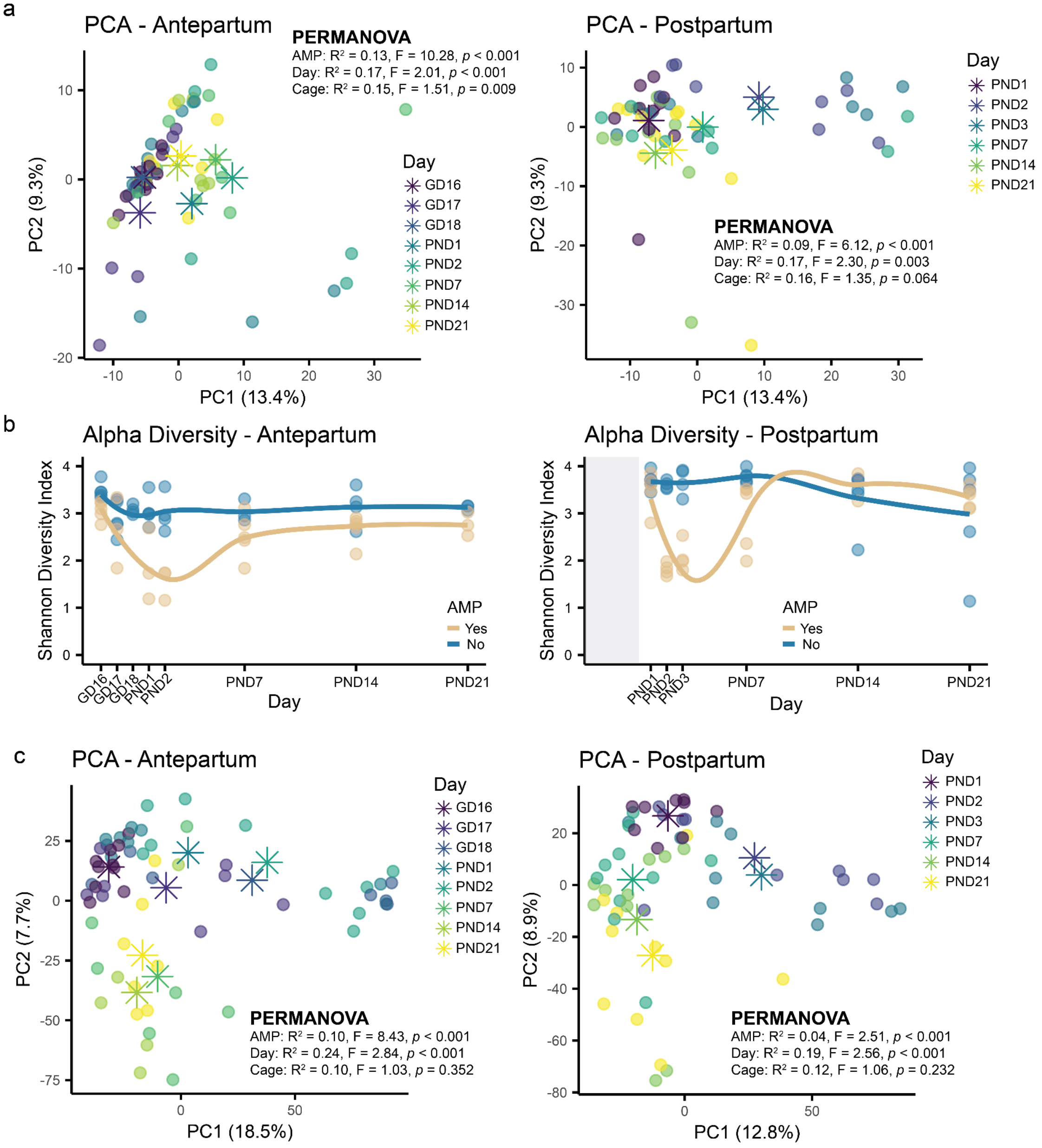

FIGURE S2

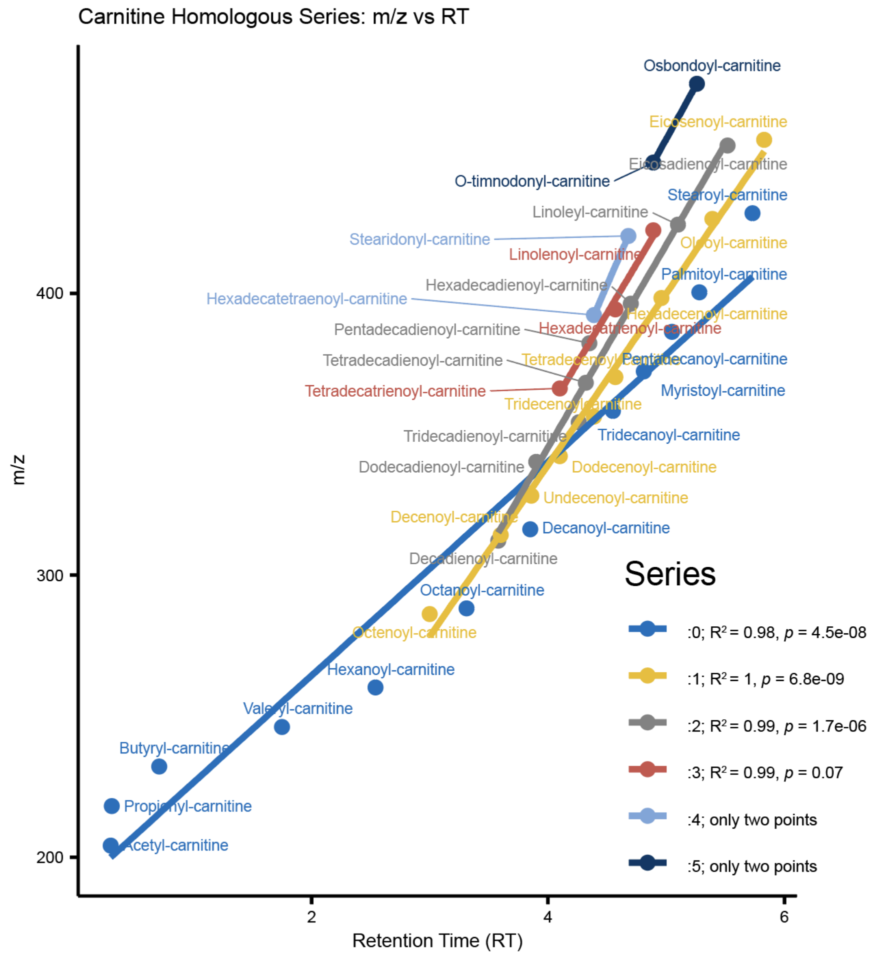

Carnitine Homologous Series: m/z vs RT
m/z
Retention Time (RT)
200
300
400
2
4
6
Osbondoyl-carnitine
Eicosenoyl-carnitine
Eicosadienoyl-carnitine
O-timnodonyl-carnitine
Stearoyl-carnitine
Linoleyl-carnitine
Stearidonyl-carnitine
Oleoyl-carnitine
Linolenoyl-carnitine
Palmitoyl-carnitine
Hexadecadienoyl-carnitine
Hexadecatetraenoyl-carnitine
Hexadecenoyl-carnitine
Pentadecadienoyl-carnitine
Hexadecatrienoyl-carnitine
Tetradecadienoyl-carnitine
Tetradecenoyl-carnitine
Pentadecanoyl-carnitine
Tetradecatrienoyl-carnitine
Myristoyl-carnitine
Tridecenoyl-carnitine
Tridecadienoyl-carnitine
Tridecanoyl-carnitine
Dodecadienoyl-carnitine
Dodecenoyl-carnitine
Undecenoyl-carnitine
Decenoyl-carnitine
Decanoyl-carnitine
Decadienoyl-carnitine
Octanoyl-carnitine
Octenoyl-carnitine
Hexanoyl-carnitine
Valeryl-carnitine
Butyryl-carnitine
Propionyl-carnitine
Acetyl-carnitine
Series
:0; R² = 0.98, p = 4.5e-08
:1; R² = 1, p = 6.8e-09
:2; R² = 0.99, p = 1.7e-06
:3; R² = 0.99, p = 0.07
:4; only two points
:5; only two points

FIGURE S3

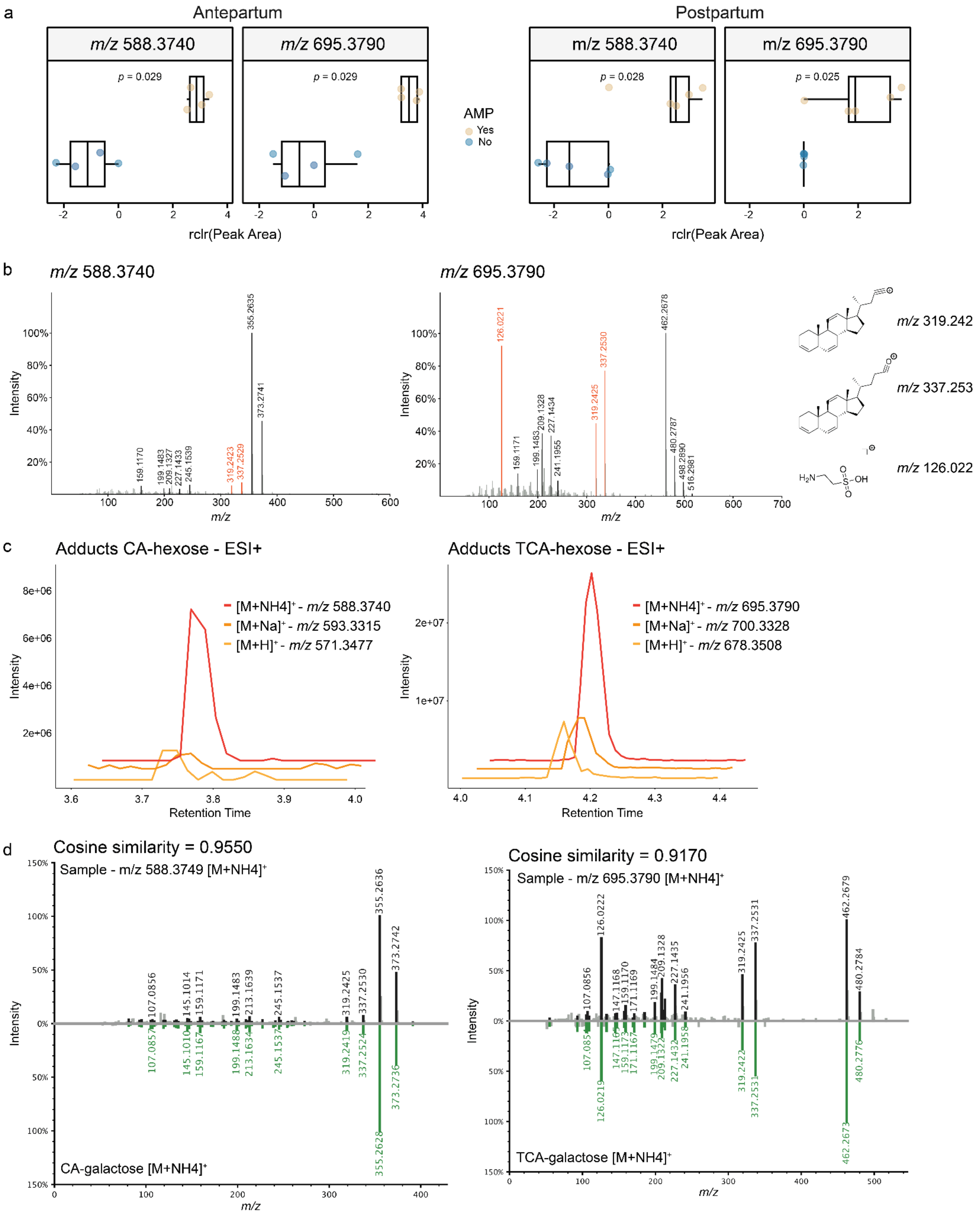

a
Antepartum
Postpartum
m/z 588.3740
m/z 695.3790
p = 0.029
p = 0.028
p = 0.025
AMP
Yes
No
rclr(Peak Area)
b
m/z 588.3740
m/z 695.3790
Intensity
m/z
m/z 319.242
m/z 337.253
m/z 126.022
c
Adducts CA-hexose - ESI+
Adducts TCA-hexose - ESI+
[M+NH4]+ - m/z 588.3740
[M+Na]+ - m/z 593.3315
[M+H]+ - m/z 571.3477
[M+NH4]+ - m/z 695.3790
[M+Na]+ - m/z 700.3328
[M+H]+ - m/z 678.3508
Retention Time
d
Cosine similarity = 0.9550
Sample - m/z 588.3749 [M+NH4]+
CA-galactose [M+NH4]+
Cosine similarity = 0.9170
Sample - m/z 695.3790 [M+NH4]+
TCA-galactose [M+NH4]+

Supplementary figure captions

Supplementary Figure 1. Gut microbiome and metabolome time-dependent change

(a) PCA of all fecal microbiome profiles collected either in the Antepartum or Postpartum cohort. In both datasets, a time-dependent change was observed (PERMANOVA, $p < 0.003$) in addition to the AMP effect. A small inter-animal variance was also observed, as the mice were single housed. Asterisks in PCA score plots represent group centroids. **(b)** Longitudinal alpha diversity analysis (Shannon diversity index). A reduced alpha diversity was observed in correspondence of AMP treatment, which was then recovered at later timepoints. **(c)** PCA of all fecal metabolic profiles collected either in the Antepartum or Postpartum cohort. A time-dependent change of the metabolomes was observed (PERMANOVA, $p < 0.001$) in addition to the AMP effect. Asterisks in PCA score plots represent group centroids. Abbreviations: AMP, ampicillin; GD, gestational day; PND, postnatal day.

Supplementary Figure 2. Carnitine homologous series

Correlation of parent ion masses (*m/z*) to retention time of annotated carnitines via library matching categorized by their degree of unsaturation (number of double bonds). P values were calculated via linear regressions. As the acyl chain length increases, the hydrophobicity increases, resulting in a later elution time, as expected in reverse-phase liquid chromatography.

Supplementary Figure 3. Putative identification of uncharacterized bile acids spectra

(a) Two metabolic features of interest were consistently enriched in animals receiving AMP across the two cohorts. **(b)** MS/MS spectra of the two features were unknown bile acids. Given the presence of the diagnostic ions for trihydroxylated bile acids (*m/z* 319.242 and *m/z* 337.253) and taurine diagnostic ion (*m/z* 126.022) in one of the two, they were putatively annotated as conjugated forms of cholic and taurocholic acid respectively. **(c)** Extracted ion chromatograms of the different ion adducts related to *m/z* 588.3740 and *m/z* 695.3790. Both features of interest appeared to be ammonium adducts, as both the proton and sodium adducts were detectable at the same retention time but they did not trigger MS/MS acquisition due to low abundance. **(d)** MS/MS spectral matching of the two features of interest to the generated synthetic standards of cholic acid-galactose and taurocholic acid-galactose.
