# Supplementary figures and images for: Perinatal ampicillin exposure alters murine maternal fecal bile acid and acylcarnitine profiles
Source: Gut Microbes. 2026 Jun 29;18(1):2690698. doi: 10.1080/19490976.2026.2690698 (PMC13321893; doi:10.1080/19490976.2026.2690698)

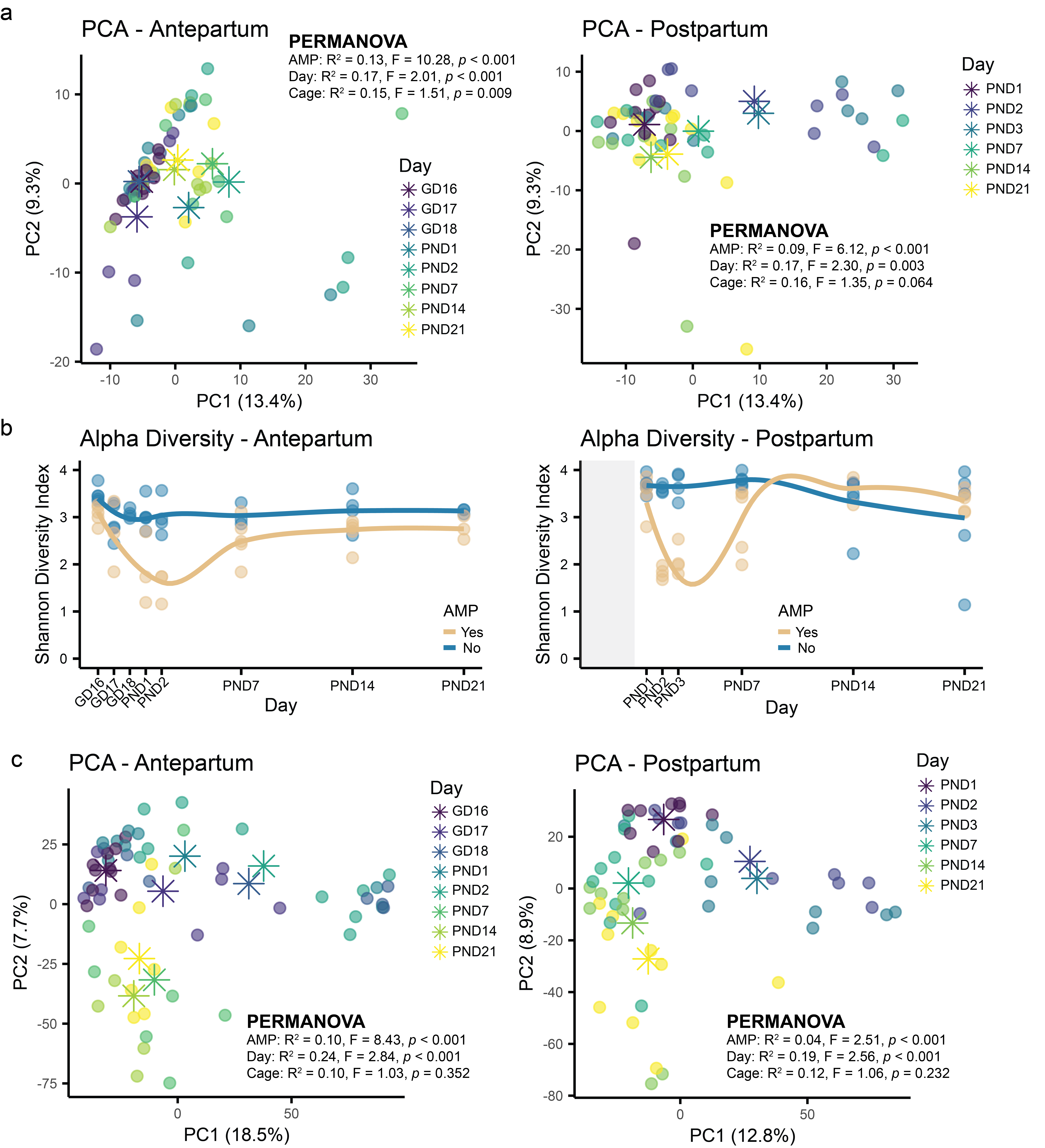

Supplement: FigureS1.png [file KGMI_A_2690698_SM2874.png]

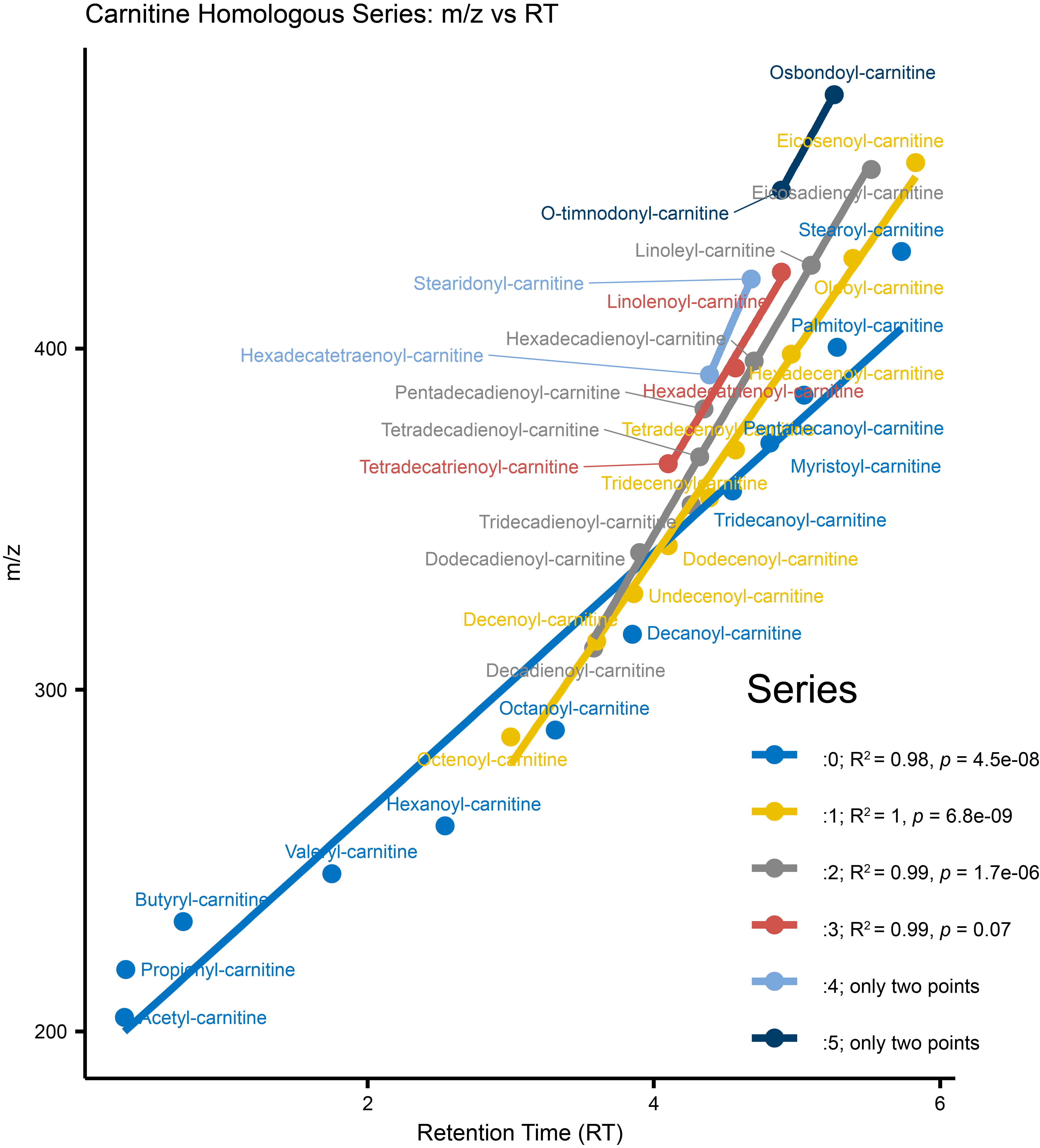

Supplement: FigureS2.png [file KGMI_A_2690698_SM2877.png]
